# MDSC in Mice and Men: Mechanisms of Immunosuppression in Cancer

**DOI:** 10.3390/jcm10132872

**Published:** 2021-06-28

**Authors:** Christophe Vanhaver, Pierre van der Bruggen, Annika M. Bruger

**Affiliations:** 1 De Duve Institute, Université Catholique de Louvain, Avenue Hippocrate 74, 1200 Brussels, Belgium; pierre.vanderbruggen@uclouvain.be; 2 WELBIO, Avenue Hippocrate 74, 1200 Brussels, Belgium

**Keywords:** MDSCs, myeloid-derived suppressor cells, T-cells, human, mouse, immunosuppressive assays, cancer biology, immunotherapy

## Abstract

Myeloid-derived suppressor cells (MDSCs) expand during pathological conditions in both humans and mice and their presence is linked to poor clinical outcomes for cancer patients. Studying MDSC immunosuppression is restricted by MDSCs’ rarity, short lifespan, heterogeneity, poor viability after freezing and the lack of MDSC-specific markers. In this review, we will compare identification and isolation strategies for human and murine MDSCs. We will also assess what direct and indirect immunosuppressive mechanisms have been attributed to MDSCs. While some immunosuppressive mechanisms are well-documented in mice, e.g., generation of ROS, direct evidence is still lacking in humans. In future, bulk or single-cell genomics could elucidate which phenotypic and functional phenotypes MDSCs adopt in particular microenvironments and help to identify potential targets for therapy.

## 1. Introduction

According to the *seed and soil* theory by Paget, the tumor microenvironment (TME) plays a major role in cancer progression [1]. Within the TME, myeloid-derived suppressor cells (MDSCs) are a rare group of myeloid cells with immunosuppressive capacities that accumulate in individuals with conditions that include cancer, but also sepsis or chronic infection [2]. While MDSCs were first described as immature or progenitor cells, recent studies extended the definition to include mature subpopulations [3,4,5]. Classically, MDSCs are divided into monocytic MDSCs (M-MDSCs) and polymorphonuclear or granulocytic MDSCs (PMN- or G-MDSCs), according to their lineage and phenotype. In humans, early stage MDSCs (E-MDSCs) represent a third progenitor subset that lacks monocytic or and granulocytic markers [6,7,8].

In humans, increased MDSC levels are correlated to poor prognoses for disease outcome, and ablating MDSCs in mice resulted in improved tumor control [9,10,11]. Thus, MDSCs are good potential predictors for clinical progression or targets for future immunotherapeutic strategies. However, current depths of knowledge and the technical sophistication with which MDSCs are approached differ between the species.

In this review, we compare isolation mechanisms for murine and human MDSCs, and the current technical limitations. We summarize the known MDSC-mediated mechanisms of suppression in each species. Finally, we propose a roadmap for future analyses of MDSCs and their functions.

## 2. Identification and Isolation

MDSCs are infrequent cell populations. They are rare in healthy individuals but expand in pathological conditions [12]. Thus, high sample volumes are required to obtain sufficient MDSCs for experimentation. Today, no MDSC-specific surface marker is known and discerning between human MDSCs and other myeloid cells relies on physical properties such as cell density or differences in the intensity of surface marker expression [6]. Identifying MDSCs correctly is further complicated by their definition as immunosuppressive cells, which requires confirmation of the cellular functions [6].

### 2.1. MDSCs in Mice

Murine MDSCs are mainly isolated from the spleen, the bone marrow (BM) or the lung [13,14,15,16]. These tissues offer the greatest yields of up to 10^7^ to 10^8^ cells [17]. It is also possible to isolate MDSCs from lymph nodes (by mechanical disruption), the liver (using Percoll), tumors (by mechanical and enzymatic disruption) or peripheral blood (by cardiac puncture) [18,19,20,21]. However, it might be necessary to combine the blood of several mice to achieve MDSC yields with which experiments can be performed. While all tissues require different pre-treatments (e.g., the spleen requires mechanical disruption and erythrocyte depletion, while the lung requires enzyme-based digestion [13,14]), all MDSCs are typically isolated using either commercial magnetic bead kits or flow cytometry [22,23,24].

**Figure 1:**
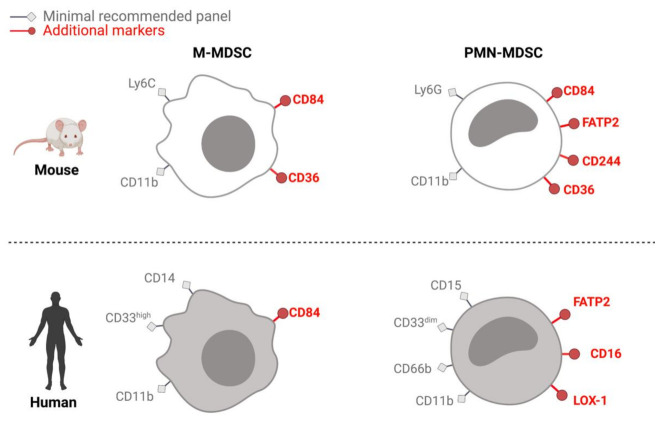
***Phenotyping M-MDSCs and PMN-MDSCs in mice and humans***. In addition to the recommended panel [6], several surface markers are used to further define M-MDSCs and PMN-MDSCs in mice and humans. CD84 is a receptor of the family of signaling lymphocytic activation molecules [26]. FATP2 stands for fatty acid transport protein 2 [27] and CD36 is a fatty acid translocase [28]. CD244 is also known as NK cell Receptor 2B4 [25]. CD16 is the Fc-receptor gamma 3 [8]. LOX-1 is a lectin-type oxidized LDL receptor [31].

Originally, all murine MDSCs were described as CD11b^+^ and Gr-1^+^. Gr-1 is composed of a subunit from the Ly6 family and cells are further classified into M- (Ly6C^+^) or PMN- (Ly6G^+^) MDSCs (Figure 1) [6,22]. To date, no E-MDSCs have been discovered in mice. CD84, CD244, fatty acid transporter protein 2 (FATP2) and CD36 have all been suggested as markers to identify M-MDSCs and PMN-MDSCs more effectively (Figure 1) [2,25,26,27,28]. For example, CD11b^+^Ly6C^low^Ly6G^high^CD244^+^ PMN-MDSCs isolated from mouse models for thymoma, melanoma or colorectal cancer suppressed CD8 T-cells more effectively than CD11b^+^Ly6C^low^Ly6G^high^CD244^−^ populations [25]. A specific marker for either M-MDSCs or PMN-MDSCs is still elusive, as all markers mentioned can be expressed by macrophages, neutrophils or dendritic cells.

The biochemical features that underlie MDSC-mediated immunosuppression-, e.g., expression of arginase-1, inducible nitric oxide synthase (iNOS) or NADPH oxidase (NOX2)-, can be used in the phenotypic analyses of MDSCs. However, these enzymes are expressed intracellularly, which means that labelling them is incompatible with isolating live cells. Furthermore, the listed enzymes are also expressed by immunosuppressive macrophages or activated neutrophils [29,30].

### 2.2. MDSCs in Humans

Human MDSCs are most commonly isolated from the peripheral blood of patients [7,26,31]. Infrequently, MDSCs are isolated from tumors following mechanical tissue disruption and enzyme-based digestion [32,33]. Additionally, the presence and intra-tumoral distribution of MDSCs (but not their immunosuppressive functions) are analyzed using immunohistochemistry [34]. In rare cases, MDSCs are isolated from the spleen [35], BM aspirates [36] or cancer ascites [37].

As access to human clinical samples can be challenging, protocols have been established to differentiate myeloid cells into MDSCs in vitro. For example, culturing peripheral blood monocytes with granulocyte-macrophage colony-stimulating factor (GM-CSF), interleukin (IL)-4 and Prostaglandin-E_2_ (PGE2) or PGE2-expressing tumor cells generates cells with immunosuppressive functions and phenotypes similar to M-MDSCs [38,39]. Similar results were obtained by culturing BM cells with granulocyte colony-stimulating factor (G-CSF), GM-CSF and IL-6 [40,41]. While generating in vitro MDSCs is enticing, myeloid cells are highly heterogeneic and plastic cells. The suitability of in vitro generated MDSCs as models for MDSCs with clinical relevance still needs to be confirmed in genetic and transcriptomic comparisons to patient-isolated MDSCs.

The 2016 seminal review by Bronte et al. clearly defined the standard minimal protocols to identify and isolate human MDSCs: M-MDSCs are CD11b^+^CD14^+^CD15^−^HLA-DR^low/−^ (Figure 1) [6]. Instead of CD11b, the myeloid marker CD33 could be used, which is expressed in high levels on monocytic cells [42]. The low expression of HLA-DR distinguishes M-MDSCs from monocytes [6]. PMN-MDSCs are CD11b^+^CD14^−^CD15^+^HLA-DR^−^[6]. CD33 is dimly expressed on granulocytic cells [42]. Either CD15 or CD66b can be used as a granulocyte marker. Phenotypically, PMN-MDSCs are currently indistinguishable from mature neutrophils. Instead, PMN-MDSCs and neutrophils are separated by density gradients [6]. Higher expression levels of CD11b and CD16 on PMN-MDSCs correlated with a stronger suppression of T-cell functions in head and neck (HN) cancer patient [8].

As in mice, although no MDSC-specific marker is known to date, several candidate surface markers that are upregulated on MDSCs have been identified (Figure 1). CD84, a gene that is upregulated on murine M- and PMN-MDSCs, is also expressed on human M-MDSCs that were generated in vitro from PBMCs [26]. Condamine et al. found that PMN-MDSCs overexpress lectin-type oxidized LDL receptor 1 (LOX-1) by comparing the transcriptomes of PMN-MDSCs and neutrophils from the blood of healthy donors, non-small cell lung carcinoma (NSCLC) patients and HN cancer patients [31]. In patients with hepatocellular carcinoma and pancreatic cancer, PMN-MDSCs expressed higher levels of LOX-1 in CD15^+^ populations compared to healthy donors [43,44]. Furthermore, Si et al. showed, using immunohistochemistry, that LOX-1-expressing tumor-associated neutrophils (TAN) from HN cancer patients also expressed arginase-1 [34]. Higher frequencies of LOX-1^+^ TAN at the tumor site correlated to decreased overall survival.

Considering the complex phenotype of human MDSCs and their rarity, it is recommended to isolate MDSCs using flow cytometry [6,45]. Magnetic selection of CD33^+^ or CD11b^+^ cells could be used as an enrichment step, but preferably not as the principal method of isolation because MDSCs are rare, and the markers used are not exclusive. No commercial isolation kits exist today for human MDSCs.

### 2.3. Common Challenges in Handling Murine and Human MDSCs

Due to their rarity, only few MDSCs are accessible for in vitro study. In patients with pathologies such as breast or colorectal cancers, PMN-MDSCs and M-MDSCs usually represent between 0.1 to 5% of PBMCs. However, in some individuals, e.g., ovarian carcinoma patients, the frequency of MDSCs is as low as 0.01% of PBMCs [7] (and own observation). Realistic MDSC yields are further limited by the constricted volumes of blood that are practically and ethically obtainable from tumor-bearing mice or cancer patients (around 2.5 and 50 mL, respectively).

MDSC samples need to be processed carefully because granulocytes are easily activated by rough handling or cold temperatures. If and how this could affect the suppressive functions of PMN-MDSCs remains unclear [45]. Careful handling of MDSC samples is especially important when the cells are isolated from tissues other than blood, as it is unknown if and how mechanical and/or enzymatical digestion affects MDSC phenotypes or functions [46].

Following isolation, MDSCs need to be immediately tested in experiments because PMN-MDSCs are cryosensitive and have short ex vivo life-spans [47,48]. For context, neutrophils typically survive for 24 h in vivo [49].

## 3. Immunosuppressive Mechanisms of MDSCs

The principal characteristic of MDSCs is their ability to suppress immune responses [6]. Consequently, and in the absence of specific markers for immunosuppressive MDSCs, a part of each MDSC sample should be tested in in vitro immunosuppression assays [45,50]. T-cells are the most documented target of MDSC-mediated immunosuppression. However, MDSCs can also affect the functions of B-cells and natural killer (NK) cells [51,52,53,54,55]. In this chapter, we will explore the known mechanisms by which MDSCs suppress T-cell functions in each species (Table 1).

### 3.1. Amino Acid Deprivation

Upon encountering their antigen, T-cells shift from a quiescent state based on oxidative respiration to an active state that relies on glycolysis, which allows for rapid growth and effector cell functions (i.e., cytokine secretion or cell lysis) [85,86]. Proliferation and effector functions require protein synthesis and, thus, activated T-cells increase their uptake of amino acids, such as glutamine [85,87,88]. T-cells detect the abundance of amino acids through mammalian target of rapamycin (mTOR) signaling pathways. An amino acid deprivation is detected through general control nonderepressible-2 kinase (GCN2) [89,90]. GCN2 is activated when it binds to uncharged transfer-RNA (tRNA). To inhibit further protein synthesis, GCN2 phosphorylates the α-subunit of eukaryotic translation initiation factor 2 (eIF2α) and, to promote catabolism and cell autophagy, GCN2 inhibits the mTOR pathway [89,90].

### 3.2. Arginine Metabolism

Arginine is a non-essential amino acid. Some pathological conditions increase the overall arginine demand, and high concentrations of arginine correlate with better survival and anti-tumor activity in cancer [91]. Low arginine levels in the TME can lead to the loss of T-cell functions, including proliferation [92,93]. Arginine is metabolized by the arginase family enzymes, which convert arginine to ornithine and urea, and nitric oxide synthase (NOS), which degrades arginine to produce nitric oxide (NO) and citrulline [91]. Both mechanisms are found in MDSCs, with the NOS pathway being particularly prominent in M-MDSCs [24]. Downstream metabolites of arginine, such as spermine or spermidine, also modulate T-cell differentiation [94].

In mice, arginine deprivation is described to be one of the main mechanisms by which MDSCs suppress T-cells. However, direct evidence of arginine depletion by MDSCs is sparse. In 3LL murine lung carcinoma, Gr-1^−^CD11b^+^ myeloid suppressor cells expressed increased levels of intra-cytosolic arginase-1 and the cationic amino acid transporter 2B, which imports extracellular L-arginine [56]. The T-cells cultured in the presence of myeloid suppressor cells proliferated more slowly, secreted fewer cytokines and downregulated the expression of the CD3ζ. Adding the arginase-1 inhibitor N-hydroxy-nor-L-arginine restored the impaired T-cell functions. COX-2 induction was responsible for the arginase-1 upregulation in Gr-1^−^ CD11b^+^ myeloid suppressor cells, and blocking COX-2 in vivo decreased the tumor volume in a mouse model of lung carcinoma [57]. However, despite being referred to as MDSCs in later reviews [58,95], the described cells expressed low levels of Gr-1, and thus, it is uncertain whether these cells may be considered as MDSCs [96]. Inhibiting arginase-1 in vivo in an NSCLC mouse model caused tumor regression and an improvement of T-cell functions [59]. In mice bearing 4T1 tumors, inhibiting iNOS and/or the arginase-1 rescued antibody-dependent cell-mediated cytotoxicity of NK cells [55]. Nonetheless, it is important to remember that arginine depletion by arginase-1 or iNOS is a mechanism shared by other myeloid cells [29,97]. Furthermore, Bian et al. showed that arginase-1 was neither expressed by nor required for MDSC-mediated immunosuppression in mouse models of lymphoma, melanoma or colorectal cancer [98]. Therefore, the depletion of arginine by MDSCs in mice requires further analysis, e.g., by specifically inhibiting arginase-1 in MDSCs if specific markers are discovered.

In humans, no direct evidence that MDSCs suppress immune responses by depleting arginine exists. PMN-MDSCs and PMN express arginase-1 in granules [60,61,62,99], and T-cells arrest their cell cycle in the absence of L-arginine or in the presence of arginase-1 [63,64]. Inhibiting arginase-1 in myeloma patients enhanced the functions of T-cells co-cultured with PMN [93]. In renal carcinoma patients, the presence of arginase-1-expressing PMN-MDSCs was correlated with decreased CD3ζ expression and impaired cytokine secretion by PBMC, which was restored by depleting PMN-MDSCs [61,62]. In NSCLC patients, the PMN-MDSCs that express arginase-1 caused a downregulation of CD3ζ in CD8^+^ T-cells and Jurkat cells during in vitro co-culture [65].

### 3.3. Cysteine Metabolism

Cysteine is a non-essential amino acid. Cysteine deprivation inhibits the formation of gluthatione, a major cellular process that prevents damage caused by reactive oxygen species (ROS) [100]. Cysteine is generated in the following two ways: (1) By converting intracellular methionine to cysteine through cystathionase. (2) By importing cystine, the oxidized form of cysteine, through the xc^−^ transporter and reducing it to cysteine with cystathionase [66,100]. However, naïve T-cells express neither the xc^−^ transporter nor cystathionase. Instead, naïve T-cells directly import cysteine through the alanine-serine-cysteine (ASC) family of transporters, which includes SLC1A4 and SLC1A5 [101]. Since naïve T-cells are unable to generate their own cysteine, they rely on antigen presenting cells to convert cystine to cysteine, which is then exported through ASC transporters.

In mice, Srivastava et al. (2010) showed that pan-MDSCs deplete cysteine from the serum [66]. MDSCs expressed high levels of the xc^−^ transporter but low levels of ASC transporters, meaning that MDSCs import extracellular cystine without releasing cysteine [66]. Cysteine sequestration by MDSCs leads to an arrest of T-cell proliferation. Supplementing a reducing agent, e.g., β-mercaptoethanol, or stable cysteine abolished the effect.

In humans, there is no evidence that either M- or PMN-MDSCs sequester cysteine. Although neither ASC nor xc^−^ transporters are expressed on naïve CD4^+^ T-cells, their expression is upregulated upon activation [102]. This suggests that activated human T-cells directly import both cystine and cysteine. Thus, the impact of MDSCs in the metabolism of cysteine and cystine in T-cells is likely negligible.

### 3.4. Tryptophan Metabolism

Tryptophan is an essential amino acid. Indoleamine 2,3-dioxygenase (IDO) or tryptophan 2,3-dioxygenase (TDO) degrade tryptophan to N-formylkynurenine [103]. T-cells and NK cells sense tryptophan deprivation or the presence of tryptophan metabolites by GCN2 (see *Amino acid deprivation introduction*), arresting T-cell proliferation, causing anergy and triggering the differentiation of CD4^+^ T-cells into regulatory T-cells (Treg) [103,104,105,106]. Tryptophan metabolites such as kynurenine, 3-hydroxykynurenine or picolinic acid also dampen T-cell and NK cell functions and proliferation [106,107], and induce Treg generation [104].

IDO-KO mice lack functional MDSCs compared to WT specimens in a model of graft versus host disease [108]. However, direct evidence of IDO expression in M- or PMN-MDSCs is still lacking. Only one study shows that murine M-MDSCs express IDO, which contributes to the suppressive effects of MDSCs on T-cells [36]. However, the MDSCs were generated in vitro from bone marrow and were never compared to *ex vivo* MDSCs phenotypically or transcriptomically.

In humans, direct evidence that MDSCs suppress T-cells by depleting tryptophan is still missing. Yu et al. (2013) observed that IDO is expressed by CD33^+^CD14^−^CD15^−^ cells, corresponding to E-MDSCs, isolated from the blood or metastasis of breast cancer patients [67]. E-MDSCs inhibited CD3^+^ T-cell proliferation in in vitro co-cultures and proliferation was restored in the presence of 1-methyl-L- tryptophan, an IDO inhibitor.

### 3.5. Generation of Oxidative Stress

Reactive oxygen species (ROS) are short-lived oxygen-containing molecules that are chemically reactive due to unpaired electrons [109]. The main enzymes involved in ROS production are NADPH oxidase (NOX) and nitric oxide (NO) synthase (NOS). NOX converts NADPH and oxygen into NADP^+^ and superoxide radicals. Superoxide dismutase (SOD) catalyzes superoxide radicals into the less reactive H_2_O_2_. NOS generates NO when it degrades L-arginine to L-citrulline. NO reacts with O_2_^−^ to form peroxynitrite ONOO^−^. ROS generate oxidative stress that damages nucleic acids, proteins and lipids, thus impairing T-cell functions and viability [109]. For example, oxidative stress downregulates or induces post-translational modifications in TCR-signaling proteins such as lymphocyte-specific protein tyrosine kinase (LCK), or CD3ζ, leading to dysfunction and/or proteasomal degradation [110,111,112]. Impaired TCR-signaling results in hyporesponsive T-cells [111,112]. Effector CD4^+^ T-cells are more sensitive to death induced by ROS than Treg [113,114].

In mice, strong evidence shows that MDSCs rely on both NOX2 and iNOS enzymes to produce ROS and inhibit T-cell or NK cell functions. Murine PMN-MDSCs express NOX2 but not iNOS, and release peroxynitrite [68]. For example, PMN-MDSCs in two different viral infection models inhibited NK cell functions by releasing ROS [72,115]. Murine M-MDSCs express iNOS but not NOX2, and produce more NO [68]. NO-based reactive molecules can cause nitration of the TCR in vivo, which leads to CD8^+^ T-cells ineffectively binding their peptide-MHC (pMHC) complex, and chemokine nitration, which impairs T-cell recruitment to the tumor site [71,116]. Blocking NOX2 (in gp91^−/−^ mice), iNOS (with L-NMMA or in iNOS^−/−^ mice) or peroxynitrite (with AT38 inhibitor or uric acid) abolishes the immunosuppressive effects of MDSCs on T-cells [68,69,70]. NK cell functions such as MHC-I-dependent cell cytotoxicity, antigen-dependent cell cytotoxicity (ADCC) and IFNγ secretion were inhibited by pan-MDSCs in a mouse model of breast cancer [55]. ADCC by NK cells was restored with L-NIL, an iNOS inhibitor, and in Nos2^−/−^ mice. L-NIL administration in EMT6-HER2, a mouse model of breast cancer, reduced tumor growth.

In humans, our knowledge of ROS-dependent immunosuppressive mechanisms is fragmentary. Upon PMA activation, CD11b^+^CD14^−^ cells from cancer patients produce higher levels of ROS compared to CD11b^+^CD14^−^ cells isolated from healthy donors [69]. Blood PMN-MDSCs in NSCLC patients express similar levels of iNOS than monocytes [65]. Co-culturing MDSCs from NSCLC patients with CD8^+^ T-cells decreased the expression of CD3ζ [65]. However, it is important to remember that ROS production and immunosuppression are not exclusive to cells traditionally defined as MDSCs. For instance, activated neutrophils from healthy donors or cancer patients suppress CD4^+^ and CD8^+^ T-cells through NOX2 and ROS [117]. Neutrophils treated with ROS inhibitors or neutrophils from patients with chronic granulomatous disease, a genetic disease that impacts NOX2 function, failed to suppress T-cells. Pan-MDSCs impaired ADCC and IFNγ secretion of NK cells in in vitro co-cultures and this inhibition was reversed with the iNOS inhibitor, L-NIL [55].

### 3.6. MDSCs and the Accumulation of Regulatory T-Cells

Besides MDSCs, other tolerogenic immune cells, such as regulatory T-cells (Treg), tightly regulate immune responses [118]. Treg regulate the activation and proliferation of NK cells and CD4^+^ and CD8^+^ T-cells [119]. The differentiation and functions of Treg are linked to the presence of immunosuppressive cytokines IL-10 and TGF-β [120]. In mice, the expression of forkhead box P3 (Foxp3) is associated with regulatory functions and, thus, defines the Treg subset. In humans, however, Foxp3^+^ expression alone is insufficient to define Treg. Foxp3 is transiently upregulated in all T-cells upon activation [121,122]. Only the stable expression of Foxp3, which is associated with DNA demethylation of a specific region in the Foxp3 locus, defines Treg [123].

In mice, several studies highlighted a direct link between murine MDSC presence and Treg differentiation, expansion and/or recruitment. For instance, CD115^+^Gr-1^+^ M-MDSCs secrete IL-10 and TGF-β in a colon-carcinoma model and induce the differentiation of Foxp3^+^ Treg in vitro and in vivo in an IL-10- and IFNγ-dependent manner [73]. In a lymphoma model, CD11b^+^ cells promoted the antigen- and arginase-dependent expansion of Foxp3^+^ Treg when co-cultured with CD4^+^ T-cells [74]. MDSCs recruit Treg in vitro and in vivo by secreting the chemokine CCR5-ligand [77]. Pan et al. showed that MDSCs promote Treg proliferation in a contact-dependent manner through CD40 and CD40L. Knocking-down CD40 or blocking CD40 with antibodies reduced the MDSC-mediated in vitro expansion of Treg by two-fold in comparison to wild-type mice [75].

In humans, as most studies use only Foxp3 to define Treg, direct evidence of Treg induction or expansion mediated by MDSCs is still lacking. M-MDSCs isolated from hepatocellular carcinoma patients secrete IL-10 and differentiate naïve CD4^+^ T-cells into Foxp3^+^CD25^+^ T-cells in in vitro cultures—an effect that was contact-dependent and blocked with anti-IL-10 antibodies [76]. The Foxp3^+^CD25^+^ T-cells that differentiated in the presence of MDSCs were immunosuppressive in cultures with autologous naïve CD4^+^ T-cells. In patients with kidney transplantations, accumulating MDSCs were correlated to increased frequencies of Foxp3-expressing T-cells [78]. When co-cultured with activated CD4^+^ T-cells, the MDSCs mediated a higher expansion of Foxp3^+^ T-cells compared to CD4^+^ T-cells [78]. In NSCLC and ovarian cancer patients, blocking CD39 and CD73 expressed on MDSCs partially decreased their immunosuppressive effect on CD8^+^ and NK cells [124,125]. CD39 and CD73 convert pro-inflammatory extracellular adenosine tri-phosphate (ATP) into adenosine (ADO), which favors Treg proliferation and CD8^+^ T-cell inhibition [126].

### 3.7. Expression of Immune Checkpoint Receptors

Immune checkpoint receptors are transmembrane proteins that act as regulators of effector T-cells in particular. Tumor cells express immune checkpoint ligands such as the Programmed Cell Death-1 (PD-1) ligand (PD-L1), which binds to PD-1 on T-cells and inhibits anti-tumor responses [127]. Anti-PD-1 or -PD-L1 recombinant antibodies, in combination with antibodies targeting immune checkpoint receptor CTLA-4, today represent the most effective cancer immunotherapy strategy. However, only up to 56% of patients with melanoma respond clinically to anti-checkpoint receptor immunotherapy and it is difficult to predict who these patients will be [128,129].

 In mice, evidence of immunosuppression mediated through direct contact between T-cells and MDSCs remains scarce. Murine M- and PMN-MDSCs express PD-L1 and it is often used to characterize MDSCs phenotypically, but not functionally [24,79,82]. In a colon-carcinoma model, M- and PMN-MDSCs expressing PD-L1 were more frequent in the tumor microenvironment compared to the peripheral blood or spleen [79]. Recently, it was discovered that myeloid cells, including M- and PMN-MDSCs, also express PD-1 [82]. While its exact role and impact on M- and PMN-MDSC function remains unclear, myeloid-specific PD-1 ablation in tumor-bearing mice more effectively decreased tumor growth compared to tumor-bearing mice with T-cell-specific PD-1 ablation [82]. The myeloid-specific PD-1 ablation (PD-1^f/fLysMcre^) prevented the accumulation of MDSCs and myeloid progenitors, while increasing the presence of effector T-cells at the tumor site. However, a checkpoint inhibitor blockade is improved when combined with the inhibition of PMN-MDSCs in HN cancer, suggesting that MDSCs impair T-cell functions with mechanisms independent of PD-L1 expression [130]. In a melanoma mouse model resistant to a combination of anti-PD-L1 and anti-CTLA-4 treatment, PMN-MDSCs induced T-cell apoptosis by expressing FasL [84]. The effect was reversed by blocking the Fas expression on CD8^+^ T-cells or by using anti-Fas antibodies.

In humans, MDSCs express PD-L1. However, it is unclear to what extent the expression of immune checkpoint receptors or death ligand contributes to MDSC-mediated immunosuppression. The frequency of PD-L1^+^ MDSCs in the blood of NSCLC patients was higher compared to that of healthy donors [81]. Interestingly, PD-L1^+^ PMN-MDSCs, but not M-MDSCs, were up to twice as frequent at the tumor site compared to the peripheral blood in patients with adenocarcinoma or squamous cell carcinoma [81]. PD-L1 levels were up to three times higher on PMN-MDSCs from melanoma patients that failed to respond to ipilimumab treatment targeting CTLA-4 than in patients that responded promptly. [9,83].

## 4. Assessing Immunosuppression In Vitro and In Vivo

As no specific MDSC marker has been identified, the phenotype alone is insufficient to define MDSCs. Therefore, a functional validation of MDSCs is required [6].

### 4.1. Immunosuppression Assays in Mice

In vitro studies often use proliferation, cytokine secretion or Treg induction to evaluate the impact of MDSCs on T-cells [56,73]. MDSC suppression can be enhanced or inhibited by adding drugs or antibodies to the co-cultures. Arginase-1 expression is analyzed using qRT-PCR or Western blot, while its activity is directly measured with urea production [59]. The presence of nitrite is measured directly through a Griess reaction and peroxynitrite using an ELISA [68,131]. The nitrosylation of proteins is assessed with specific anti-nitrotyrosine antibodies [70]. Alternatively, the suppressive activity of MDSCs isolated from mice that lack the enzymes related to ROS production is compared to that of MDSCs isolated from wildtype mice [24]. Sex, genetic background or in vitro differentiation protocols for MDSCs have all been shown to influence the immunosuppressive capacities of MDSCs [132,133,134].

In vivo studies modulate MDSC functions in ongoing disease by using genetically altered mice, small molecule inhibitors or blocking antibodies. Alternatively, MDSCs have been adoptively transferred into mice that do not harbor functional MDSCs [71,135]. The lack of MDSC-specific markers or transcription factors limits the options available to modulate MDSCs specifically in vivo. The antibodies used in depletion assays target the Gr-1 subunits, Ly6C and Ly6G. However, Ly6C targets both M-MDSCs and monocytes, and Ly6G targets both PMN-MDSCs and neutrophils. The technical limitations of depletion assays need to be considered, such as the rapid reconstitution of the depleted myeloid compartment [136,137]. Similarly, the immunosuppressive mechanisms discussed in the previous section are not exclusively employed by MDSCs. Other cells of the myeloid lineage-, e.g., tumor-associated macrophages or neutrophils, deplete amino acids, produce ROS, secrete IL-10/TGF-β or express PD-1/PD-L1. Therefore, targeting one mechanism within the myeloid lineage will target multiple subpopulations besides MDSCs [82].

An elegant approach to assess the effect of MDSCs in vivo is adoptively transferring purified MDSCs and T-cells into mice. For example, Nagaraj et al. intravenously injected OT-1-specific T-cells into naïve animals, then transferred MDSCs and, finally, immunized the mice against the relevant peptide (SIINFEKL) [71]. T-cell functions, e.g., proliferation or cytokine secretion, were impaired after a few days in the presence of functional MDSCs. In contrast, Sierra et al. loaded the SIINFEKL peptide directly onto MDSCs before transferring them into mice to analyze whether MDSCs required antigen-presentation for their suppressive activities [135]. This approach showed that the T-cells exposed to peptide-loaded MDSCs were dysfunctional compared to the T-cells exposed to peptide-loaded DC.

### 4.2. Immunosuppression Assays in Humans

T-cell proliferation or cytokine secretion assays are most commonly used to test the immunosuppressive activities of human MDSCs [50]. However, it is difficult to compare the results among different research groups because the protocols vary widely [7,50]. In all protocols, T-cells are co-cultured with MDSCs in 96-well tissue culture plates. T-cells are stimulated with anti-CD3- and anti-CD28-antibodies that are plate-bound and/or soluble. Stimulation beads are also used, but researchers should consider that myeloid cells phagocytose the beads, which could introduce bias to the assay. T-cell proliferation is measured either by tracking dye dilution or tritiated thymidine incorporation. Cytokine secretion, mostly IFNγ, IL-2, IL-10 or TGF-β, is assessed using an ELISA. Effector cells include autologous and allogeneic cells, PBMC, CD3^+^, CD4^+^, CD8^+^ and NK cells. Autologous T-cells show no allogeneic reaction to the MDSCs and might reflect physiological reactions better. However, allogeneic T-cells represent consistent responders across experiments and can be interchanged with other laboratories [45]. Other mechanisms of suppression studied in vitro include Foxp3^+^ CD4^+^ Treg induction, increased PD-1 expression, CD3ζ downregulation or the presence of nitrotyrosine in CD8^+^ T-cells [36,65,71].

Most studies rely on ex vivo samples from blood or biopsies to analyze the expression of surface markers and the presence of possible suppressive mechanisms (e.g., ROS production or Treg induction) [50,138,139]. In vivo studies in humans observe the effects of chemo- or immunotherapy on MDSCs levels or myeloid cell maturation [140]. However, as the therapies usually impact all myeloid cells, it is difficult to draw MDSC-specific conclusions. Humanized mouse models could represent an interesting surrogate to human in vivo studies. For example, NOD scid γ (NSG) mice are immunodeficient and allow human immune cells to develop after human CD34^+^ hematopoietic stem cells engraftment [141]. Moreover, NSG mice can be crossed with mice that are deficient of the proto-oncogene c-kit, a receptor tyrosine kinase [142]. The resulting NBSGW mice can then be engrafted with human hematopoietic stem cells without requiring prior irradiation. However, these mice do not support the expansion of human myeloid cells because they lack the necessary human cytokines. Human tumor cells can be injected into these mice before transferring a tumor-specific T-cell that had been cultured with pan-MDSCs or control cells [143].

## 5. Roadmap to Future

In both mice and humans, MDSCs accumulate under pathological conditions and suppress the functions of T-cells and NK cells. However, the road leading to specifically targeting MDSCs in diseases such as cancer remains long and demanding. The mechanisms behind how MDSCs accumulate are still unclear. More importantly, identifying MDSCs remains challenging due to the lack of unique markers. Finally, the suppressive mechanisms remain incompletely characterized, especially in humans, where in vivo experiments are very limited. We propose perspectives and suggestions for methods to increase the knowledge surrounding MDSCs and how this information may help to build better therapeutic approaches.

### 5.1. How to Identify MDSCs?

In both species, current M-MDSC markers are shared by monocytes and macrophages, while PMN-MDSC markers are also expressed by neutrophils. Currently, the distinction between MDSCs and other myeloid cells relies on their cell density and/or a diminished expression of markers such as HLA-DR or S100-family proteins [6,144]. However, these criteria limit the accuracy of identifying tumor-infiltrating MDSCs using immunohistochemistry.

To identify potential markers that are exclusive to MDSCs, we favor RNA sequencing approaches because they are unbiased and analyze all differentially expressed genes. RNA sequencing approaches identified LOX-1 as a potential PMN-MDSC and CD84 as a potential pan-MDSC marker in mice and humans [26,31]. While LOX-1 has gained wide-spread interest, its expression is often analyzed on whole neutrophil populations, without a distinction between high-density and light-density neutrophils. Comparing LOX-1^−^ vs. LOX-1^+^ light density neutrophils from the same patient could further validate this surface protein as a sufficient and unique marker to distinguish PMN-MDSCs from light- and high-density neutrophils [17]. CD84 was identified by single-cell RNA sequencing from whole murine CD11b^+^Gr-1^+^ cell populations. PMN-MDSCs were identified as a cluster of arginase-2- and IL-1β-positive PMN. CD84 was identified as a human pan-MDSC marker from in vitro-generated MDSCs. However, because the data was not compared to pure or clinical samples, it is likely that, similar to LOX-1, CD84 is not exclusive to MDSCs [25]. Nevertheless, approaches relying on bioinformatics are promising and should be repeated against control cells. For instance, new biomarkers related to MDSCs could be found by comparing the transcriptome of MDSCs and other myeloid cells from the same donor or from patients and healthy individuals. In some pathological conditions, cells that express markers used to identify MDSCs but that fail to suppress also accumulate [6,145]. Comparing the differentially expressed genes in the “MDSC-like” cells to those expressed by suppressive MDSCs would be valuable to identifying MDSC-specific markers.

### 5.2. MDSCs: State of Activation or Distinct Populations?

As MDSCs share many phenotypic and functional elements with neutrophils and monocytic cells, uncertainty remains about whether MDSCs can be considered as distinct populations. Tumor-associated macrophages and tumor-associated neutrophils, in particular, display immunosuppressive mechanisms and favor cancer progression [29,30]. It raises the possibility that PMN-MDSCs, for example, are pathologically activated neutrophils [18,29,146,147]. Originally, M- and PMN-MDSCs were characterized by their immature state to distinguish them from mature and terminally differentiated myeloid cells [148]. However, light density neutrophils that highly express the maturity marker CD16, show higher suppressive capacities than immature cells [8].

 Wagner et al. provide a guideline of how single-cell genomic approaches such as single-cell RNA-sequencing (scRNA-seq) could elucidate myeloid cell subpopulations, their responses to stimuli, their spatial context and their developmental state in greater detail [149]. New subsets of monocytes and dendritic cells in human blood have been identified already using scRNA-seq [150]. Additionally, scRNA-seq can be combined with other techniques, such as cytometry by time-of-flight (CyTOF), to gather information on the origin and lineage of MDSCs. This approach has already been used to characterize subpopulations of dendritic cells [151]. Alternatively, targeting gene expression in single cells can provide enhanced information on specific gene sets at a lower sequencing depth [152,153]. Finally, scRNA-seq could shed light on the heterogeneity inherent to MDSCs: Are MDSCs composed of distinct subpopulations, or do they fall within a plastic spectrum of activation states?

### 5.3. Relevance of MDSCs in the Clinic—Potential Therapeutic Targets?

Regardless of whether MDSCs are a distinct myeloid cell subpopulation or rather represent a state of myeloid cell activation, MDSCs are attractive therapeutic targets, as they are more frequent in patients with diverse conditions and are highly immunosuppressive.

To validate potential antibodies or drugs against MDSCs, mouse models, especially humanized mice, hold great potential and will be crucial. Not only could they enable the study of potential immunotherapies targeted at MDSCs, but they could also help understand how MDSCs emerge in response to tumors and how tumors maintain their recruitment. Studying, for example, the role of PGE2 in the initial recruitment of MDSCs to a tumor site or their maintenance in humanized mice could be interesting. However, human MDSCs fail to develop in NSG mice because the mice lack the cytokines required to maintain human myeloid cells (e.g., GM-CSF or M-CSF). The MITRG or MISTRG mice strains could support human myeloid cells because the mice express human GM-CSF, IL-3, M-CSF, thrombopoietin and SIRPα on a *Rag2^−/−^Il2rg^−/−^* background [154].

In parallel, clinical research on MDSCs will benefit from the efforts to harmonize identification strategies, isolation methods and functional assays. Cassetta et al. demonstrated that small variations in the processing of patient samples impact the frequency of MDSCs that are observable and isolatable [7]. The most difficult aspect are functional assays that are used to assess immunosuppressive capacities of isolated MDSCs. As protocols used in these assays are prone to high variations, the comparison of results among different research groups might be challenging. We previously proposed a protocol to isolate MDSCs and test their suppressive activity in proliferation assays with allogeneic T-cells that can be frozen and shared to reduce some variability inherent to these assays [45]. However, while T-cell proliferation assays remain the most common test to assess the MDSC-mediated suppression of T-cell function, it is important to consider that *ex vivo* MDSCs only survive for the first couple of days of the experiment.

 Besides technical considerations, it is important to remember that most patient MDSCs are identified and isolated from blood. However, MDSCs are thought to exert their immunosuppressive functions most at the site of disease, for example within the tumor. MDSCs samples from blood might not fully represent the MDSCs in the TME phenotypically or functionally. Thus, interesting potential markers or functions could be missed [81,155,156,157]. Very few groups isolate MDSCs directly from tumor samples. The immunohistochemistry of tumor-MDSC-T-cell interactions will provide much information, especially if MDSC-specific markers can be identified by RNA sequencing. *Si* et al. used the immunohistochemistry of HN tumor samples to correlate the expression of LOX-1 on PMN-MDSCs and their proximity to T-cells in the TME with long-term patient survival [34]. Additionally, emerging techniques such as spatial transcriptomic or multimodal omics with the CODEX system may also further illuminate the phenotypes and functions of MDSCs in tumors [158,159].

In addition to the humanized mice, patient-derived organoids could be a creative model that accounts for longer time-courses and more diverse cell–cell interactions [160,161]. However, in vitro organoids often aim to represent the TME only. This would disregard potential interactions with other compartments, e.g., lymph nodes [162]. Multi-compartment microfluidic chips that connect two ex vivo tissues might overcome this challenge and provide more information about MDSCs and the wider TME [163].

Although these methods are in their infancy, we think that targeting MDSCs in human diseases, such as cancer, holds great therapeutic potential. MDSC-specific delivery of chemotherapeutic reagents is very interesting. For example, RNA aptamers conjugated with doxorubicin [164] and lipid nanocapsules loaded with gemcitabine [165,166] specifically targeted tumor-associated myeloid cells and reduced the tumor burden of treated mice. Additionally, genes of tumor-associated myeloid cells were silenced in vivo with functionalized dendrimers [167]. Finally, studies such as the myeloid-specific ablation of PD-1 in mice or correlations of MDSCs frequencies in patients with response rates to checkpoint inhibitor targeting, indicate future paths to developing more efficient and personalized immunotherapies.

## Figures and Tables

**Table 1:** Summary of immunosuppressive mechanisms related to MDSCs.

Mechanisms of Suppression		Mouse	Ref.	Human	Ref.
**Amino acid** **depletion**	L-Arginine	PMN-MDSCs express arginase-1 ^a^. Through arginase-1, PMN-MDSCs inhibit proliferation, cytokine secretion and decrease CD3ζ expression in T-cells during in vitro co-culture.	[56,57,58,59]	*PMN-MDSCs and activated PMN express arginase-1. T-cells activated in the presence of arginase-1 or L-arginine-depleted medium arrest the cell-cycle.*	[60,61,62,63,64]
		*Tumors regress* in vivo *after inhibition of arginase-1.*	[61]	*The presence of arginase-1-expressing PMN-MDSCs in the blood of cancer patients is correlated to PBMCs losing CD3ζ expression.*	[58,59,62,65]
	L-Cysteine	M- and PMN-MDSCs sequester cysteine. Cysteine concentrations are lower in the serum of tumor-bearing mice.	[66]		
	L-Tryptophan	*Bone marrow* (in vitro)-*derived M-MDSCs express IDO. Inhibiting IDO restores T-cell proliferation.*	[36]	*E-MDSCs isolated from blood and metastasis of cancer patients express IDO. IDO expression is correlated with Foxp3^+^ T-cell expansion.*	[67]
**Oxidative stress**		PMN-MDSCs produce ROS (peroxynitrite) by NOX2. M-MDSCs produce NO by iNOS. Inhibiting ROS or ROS-producing enzyme blocks suppressive capacities of M- and PMN-MDSCs.	[68,69,70,71]	*PMN-MDSCs isolated from cancer patients produce ROS and increased levels of nitrotyrosine are present in CD8^+^ T-cell.*	[66,68]
		M- and PMN-MDSCs mediate nitration of the TCR in vivo and in vitro, which disrupts pMHC binding to CD8^+^ T-cells.	[71]	*T-cells co-cultured with iNOS-expressing PMN-MDSCs isolated from cancer patients express diminished levels of CD3ζ.*	[65]
		M- and PMN-MDSCs produce ROS, which inhibits proliferation, cytokine secretion, ADCC and granzyme-B production by NK cells.	[72]	M- and PMN-MDSCs from cancer patients inhibit cytokine secretion and ADCC of NK cell through NO production by iNOS.	[55]
**Treg**		M-MDSCs produce IL-10 and TGF-β. Foxp3^+^ Treg expand in vivo and in vitro in IL-10-, TGF-β-, arginase- and CD40L-dependant manners.	[73,74,75]	M-MDSCs induce Treg in an IL-10-dependant manner.	[76]
		M-MDSCs produce CCR5-ligand in vivo and in vitro, which recruits of Treg.	[77]	M- and PMN-MDSCs that express CD39 and CD73 generate adenosine and inhibit T-cell and NK cell proliferation and cytokine secretion.	[75,76]
				*Accumulation of M-MDSCs correlate with increased frequency of circulating Foxp3^+^ T-cells in renal transplant patients.*	[78]
**Direct MDSC/** **T-cell** **interaction**	Immune checkpoint blockade	*PD-L1^+^ M- and PMN-MDSCs present at higher frequency in tumor microenvironment compared to peripheral blood.*	[79,80]	*PD-L1^+^ M- and PMN-MDSCs accumulate in blood of cancer patients. PD-L1^+^ PMN-MDSCs accumulate in TME.*	[81]
		*Myeloid-specific ablation of PD-1 decreases tumor growth.*	[82]	*PMN-MDSCs from non-responding cancer patients (ipilimumab) express more PD-L1^+^.*	[83]
	Cell death ligand	FasL-expressing PMN-MDSCs induce CD8^+^ T-cell apoptosis.	[84]		

Direct causality of MDSCs shown – *Indirect observation or correlation*. ^a^ Rodriguez et al. (2008) initially defined MDSCs as mature Gr-1^−^CD11b^+^ myeloid suppressor cells before using MDSC terminology in later reviews. Miret et al. (2019) used only anti-CD11b to isolate MDSCs. IDO: indoleamine 2,3-dioxygenase, ROS: reactive oxygen species, NO: nitric oxide, iNOS: inducible nitric oxide synthase, NOX2: NADPH oxidase 2, TCR: T-cell receptor, pMHC: peptide-MHC complex, ADCC: antigen-dependent cell cytotoxicity, FasL: Fas ligand, PD-L1: programmed-death ligand 1, TME: tumor microenvironment, Treg: regulatory T-cells.

## Data Availability

Not applicable.
